# MicroRNA 214 Is a Potential Regulator of Thyroid Hormone Levels in the Mouse Heart Following Myocardial Infarction, by Targeting the Thyroid-Hormone-Inactivating Enzyme Deiodinase Type III

**DOI:** 10.3389/fendo.2016.00022

**Published:** 2016-03-09

**Authors:** Rob Janssen, Marian J. Zuidwijk, Alice Muller, Alain van Mil, Ellen Dirkx, Cees B. M. Oudejans, Walter J. Paulus, Warner S. Simonides

**Affiliations:** ^1^Department of Physiology, Institute for Cardiovascular Research, VU University Medical Center, Amsterdam, Netherlands; ^2^Department of Cardiology, Division of Heart and Lungs, University Medical Center Utrecht, Utrecht, Netherlands; ^3^Department of Cardiology, Faculty of Health, Medicine and Life Sciences, CARIM School for Cardiovascular Diseases, Maastricht University, Maastricht, Netherlands; ^4^Department of Clinical Chemistry, Institute for Cardiovascular Research, VU University Medical Center, Amsterdam, Netherlands

**Keywords:** thyroid hormone metabolism, Dio3, SECIS, microRNA, myocardial infarction

## Abstract

Cardiac thyroid-hormone signaling is a critical determinant of cellular metabolism and function in health and disease. A local hypothyroid condition within the failing heart in rodents has been associated with the re-expression of the fetally expressed thyroid-hormone-inactivating enzyme deiodinase type III (Dio3). While this enzyme emerges as a common denominator in the development of heart failure, the mechanism underlying its regulation remains largely unclear. In the present study, we investigated the involvement of microRNAs (miRNAs) in the regulation of *Dio3* mRNA expression in the remodeling left ventricle (LV) of the mouse heart following myocardial infarction (MI). *In silico* analysis indicated that of the miRNAs that are differentially expressed in the post-MI heart, miR-214 has the highest potential to target *Dio3* mRNA. In accordance, a luciferase reporter assay, including the full-length 3′UTR of mouse *Dio3* mRNA, showed a 30% suppression of luciferase activity by miR-214. In the post-MI mouse heart, miR-214 and Dio3 protein were shown to be co-expressed in cardiomyocytes, while time-course analysis revealed that *Dio3* mRNA expression precedes miR-214 expression in the post-MI LV. This suggests that a Dio3-induced decrease of T3 levels is involved in the induction of miR-214, which was supported by the finding that cardiac miR-214 expression is down regulated by T3 in mice. *In vitro* analysis of human *DIO3* mRNA furthermore showed that miR-214 is able to suppress both mRNA and protein expression. *Dio3* mRNA is a target of miR-214 and the Dio3-dependent stimulation of miR-214 expression in post-MI cardiomyocytes supports the involvement of a negative feedback mechanism regulating *Dio3* expression.

## Introduction

Myocardial infarction (MI) is one of the major causes of heart failure in Western society (1). The sudden interruption of blood flow results in cardiomyocyte death by necrosis and subsequent fibrosis of the affected part of the left ventricle (LV). The associated increase in cardiac load of the non-infarcted region of the heart triggers functional and structural remodeling of the spared myocardium aimed at maintaining cardiac output (2, 3). The process of remodeling includes hypertrophy of cardiomyocytes, i.e., primarily lengthening by addition of sarcomeres in series, and marked changes in gene expression, including re-expression of part of the fetal gene program. Progressive remodeling, LV dilation, and the associated increase in wall stress often culminate in chronic heart failure. The underlying changes in gene expression are mediated by a multitude of signal transduction pathways involving transcriptional regulation, as well as non-coding transcripts, such as microRNAs (miRNAs), that affect mRNA stability or translation efficiency (3–6).

One of the genes that is re-expressed as part of the fetal gene program in various models of cardiac hypertrophy and heart failure is the thyroid-hormone-inactivating enzyme, deiodinase type III (Dio3) (7–11). Dio3 belongs to a family of 25 so called selenoproteins that require a mRNA stem loop structure located in the 3′UTR region, termed a selenocysteine insertion sequence (SECIS) element, for incorporation of a selenocysteine critical for enzymatic activity [reviewed by Berry et al. (12)]. Dio3 converts the active form of thyroid hormone, triiodothyronine (T3), to the inactive metabolite diiodothyronine (3,3′-T2) [reviewed by Gereben et al. (13)]. In both mouse and rat models of ventricular failure, the re-expression of *Dio3* in the remodeling LV is associated with a ~50% reduction of tissue T3 levels and a similarly reduced T3 transcriptional activity in cardiomyocytes *in vivo* (8, 9). This local impairment of T3 signaling is suggested to play a role in the development of cardiac dysfunction given the fact that T3 is an important regulator of cardiac contractility and metabolism [reviewed by Klein and Ojamaa (14)].

Expression of Dio3 has been shown to be regulated by transforming growth factor β (Tgfβ), mitogen-activated protein kinases (Mapk), sonic hedgehog (Shh), and hypoxia-inducible factor-1α (Hif-1α) (9, 15, 16). Although the signaling cascades involving these factors are all implicated in cardiac remodeling [reviewed by Pol et al. (17)], none of these have as yet been shown to be involved in the re-expression of *Dio3* in the heart. Given the strict spatiotemporal regulation of Dio3 activity during fetal development, such as in the retina and cochlea (18, 19), *Dio3* expression is considered a likely candidate for regulation by miRNAs.

MicroRNAs are evolutionary conserved small non-coding RNA molecules of approximately 22 nucleotides long that are encoded within the genomes of almost all eukaryotes. In general, miRNAs post-transcriptionally regulate protein synthesis through base pairing with sufficiently complementary sequences in the 3′-untranslated region (3′UTR) of target mRNAs (20). Recent studies have shown an important role for miRNAs in the regulation of cardiac gene expression by virtue of their ability to induce degradation of specific target mRNAs or reduce the efficiency of translation (5, 21). Although up or down regulation of miRNAs is generally considered to fine-tune gene expression, several studies have provided insight into the critical roles of individual miRNAs in the different processes associated with development and disease, and in particular the progression of heart failure (21–24).

In this study, we therefore investigated whether miRNAs play a role in the regulation of *Dio3* expression in the remodeling heart, using the post-MI model in the mouse. In a previous study using this model, we identified miRNAs that were differentially modulated (25). *In silico* analysis performed in the present study identified only one of these miRNAs as potentially targeting *Dio3*. However, this miR-214-3p (hereafter referred to as miR-214) was upregulated in MI, implying suppression of *Dio3* expression. Upregulated expression of the highly conserved miR-214 in cardiac hypertrophy and heart failure has been demonstrated in several cardiac miRNA profiling studies in both human and experimental animal models (21, 24–26). It has been shown that elevated expression levels of miR-214 induce hypertrophy (21, 27) by suppressing peroxisome proliferator-activated receptor δ (*Ppar*δ) (27). In contrast to the apparently unfavorable effects of miR-214 in cardiac hypertrophy and heart failure, miR-214 has been shown to protect the heart from ischemic injury in a knock-out model, by suppressing the expression of the sodium calcium exchanger *Ncx1* and consequently preventing Ca^2+^ overload of cardiomyocytes and subsequent cell death (22).

Given the observed role of miR-214 in cardiac remodeling, we tested its possible relevance for the regulation of Dio3 expression. Our results show that *Dio3* is a target of miR-214 and suggest that a negative feedback mechanism exists, in which the upregulation of miR-214 dampens the MI-induced upregulation of *Dio3*, limiting the reduction of cardiac T3 signaling.

## Materials and Methods

### Prediction of miRNAs Targeting Dio3

Putative miRNAs targeting *Dio3* were identified by combining the results of different prediction databases: Targetscan v5.2, PicTar v03-2007, mirDB, and MicroCosm v5. Only miRNAs that were called by all four databases were considered for further analysis.

### Luciferase Reporter Experiments

Approximately 7.5 × 10^4^ HEK293 cells were seeded per well in 48-well plates in growth medium (DMEM containing 10% FBS, 1% NEAA, 1% sodium pyruvate, and 1% P/S). The next day, at 50% confluence, medium was changed to 200 μl DMEM per well. To increase the accuracy of the determination of miR-214-dependent luciferase activity, cells were transfected with 50 ng of a single dual-luciferase reporter plasmid containing the *Dio3* 3′-UTR downstream of the *Renilla luciferase* gene (*RLuc*). Firefly luciferase activity (*FLuc*) was used to correct for differences in transfection efficiency. To mimic or inhibit miRNA action, chemically modified double-stranded RNA molecules were added. These included Pre-miR-214 (100 nM); a combination of Pre-miR-214 (50 nM) and Anti-miR-214 (50 nM); or negative control miR#1 (100 nM) (Ambion, Foster City, CA, USA). Both plasmids and miRNAs were transfected using Lipofectamine 2000 (Invitrogen, Carlsbad, CA, USA). After 6 h, the transfection medium was replaced by growth medium. Forty-eight hours after the start of transfection, medium was removed and cells were lysed for 15 min in 50 μl lysis buffer (Promega, Madison, WI, USA) and the activity of *RLuc* and *FLuc* luciferase activity was assessed using the Dual-Luciferase^®^Reporter Assay System (Promega, Madison, WI, USA), according to manufacturer’s instructions.

### Mouse Model of Myocardial Infarction

A total of 12 C57Bl/6J mice (Harlan, 10–12 weeks old) of either sex were randomly assigned to the sham-operated group or MI group, weighed and anesthetized. MI was induced by ligation of the left coronary artery (LAD) and echocardiography measurements were performed as previously described (28, 29). Briefly, under anesthesia (2.5% isoflurane in a mixture of air and O_2_), a thoracotomy was performed at the fourth left intercostal space and the LAD was permanently ligated. The occlusion was confirmed by the slight change in color of the anterior wall of the LV downstream of the ligature. Sham-operated mice underwent the same procedure except for the occlusion of the LAD. After 1 week, echocardiography was used to establish the reduced contraction of the LV free wall. LV tissue from sham animals and non-infarcted, i.e., spared LV tissue from MI animals was collected, frozen in liquid nitrogen and stored at −80°C. In addition, we used LV tissue (post-MI days 3, 5, 7, 14, 35, or 56) from previous experiments (8) that were performed according to above-described protocol.

### Mouse Model of Hyperthyroidism

Left-ventricular samples from a previous study were used to assess miR-214 expression (30). Briefly, in this study two groups of six male C57BL/6 mice (age 10–12 weeks, Charles River) were used. Both groups were allowed *ad libitum* access to food containing propylthiouracil (PTU) (Teklad + 0.15% PTU) for 41 days to induce hypothyroidism. Animals were maintained on this diet and six mice were injected intraperitoneally with a supra-physiological dose of 5 μg T3 in 20 μl saline (corresponding to 0.21–0.24 μg T3/g BW) at days 42, 43, and 44, while the remaining six mice were injected with 20 μl saline. Animals were sacrificed at day 45. Left-ventricular tissue from hypothyroid and hyperthyroid mice was collected, frozen in liquid nitrogen and stored at −80°C until analysis.

All experiments performed on animals complied with the Guide for Care and Use of Laboratory Animals of the National Institutes of Health (NIH Publication no. 86–23, revised 1996) and were approved by the Institutional Animal Care and Use Committees of VU University Medical Center Amsterdam.

### Quantitative PCR and miRNA Analysis

Total RNA was isolated from LV tissue of sham, post-MI, hypo-, and hyperthyroid mice using mirVana PARIS kit for miRNA (Ambion, Foster City, CA, USA) or TriPure for mRNA (Roche, Basel, Switzerland) and treated with DNaseI (Qiagen, Venlo, The Netherlands) to remove remnants of genomic DNA, followed by reverse transcription reaction with either Cloned AMV First Strand synthesis kit (Invitrogen, Carlsbad, CA, USA) or with miRCURY LNA Universal cDNA synthesis kit II (Exiqon, Vedbæk, Denmark). Expression levels of atrial natriuretic factor (*Anf*), MHCα (*Myh6*), MHCβ (*Myh7*), and *Dio3* mRNA were determined by qPCR using specific primers (Table S1 in Supplementary Material) and standard cycle parameters on an Applied Biosystems model 7500 (Applied Biosystems, Foster City, CA, USA) with hypoxanthine-guanine-phosphoribosyl-transferase (*Hprt*) as correction factor. Expression levels of miR-214-3p were analyzed using miRCURY LNA miRNA primers and normalized against U6 snRNA (Exiqon, Vedbæk, Denmark).

### *In Situ* Hybridization

Before tissue sectioning, all equipment was heat-treated or cleaned with RNAzap (Sigma-Aldrich, St. Louis, MO, USA) to eliminate RNase contamination. Paraformaldehyde-fixed and paraffin-embedded sections (6 μm) were deparaffinized according to the manufacturer’s instructions for *in situ* hybridization (Exiqon, Vedbæk, Denmark) (31). Sections were incubated with 5 μg/ml proteinase-K for 30 min at 37°C, washed in PBS and immediately dehydrated using solutions with increasing concentration of ethanol. When dry, slides were placed in a specially designed hybridization chamber. To prepare the double-digoxigenin (DIG) miRCURY LNA microRNA Detection probe (Exiqon, Vedbæk, Denmark), an aliquot was heated to 90°C for 4 min and diluted to 40 nM in ISH buffer (Exiqon, Vedbæk, Denmark). Approximately 50 μl was applied directly on the dried tissue section. The sealed hybridization chamber was placed in an oven at an optimized temperature of 57°C for 1 h. Slides were then placed in a glass jar containing 5 × SSC at room temperature. Stringent washes were performed in pre-heated SSC buffers (hybridization temperature, 57°C) of 5 min each: once in 5 × SSC, twice in 1 × SSC, and twice in 0.2 × SSC. Next, the slides were placed in 0.2 × SSC at room temperature and transferred to a glass jar containing PBS containing 0.1% Tween-20. To prevent non-specific binding of anti-DIG, sections were blocked using PBS containing 0.1% Tween-20 and 2% goat serum for 15 min at room temperature. Alkaline-phosphatase (AP) conjugated anti-DIG (Roche, Basel, Switzerland) was diluted 1:500 in blocking buffer and incubated for 2 h at room temperature. Slides were washed three times with PBS containing 0.1% Tween-20. Alkaline-phosphatase substrate [3.4 μl NBT, 3.5 μl BCIP, 2.4 μl 1.25 mM Levamisole, 5 μl 10% Tween-20 in 1 ml substrate solution: 0.1M Tris pH 9.5, 0.1M NaCl, 50 mM MgCl_2_ (32)] was applied at room temperature and replaced with fresh solution after 1 h and incubated overnight. Placing the slides two times in PBS for 5 min stopped the enzymatic reaction. For counterstaining the sections, we applied 100 μl Nuclear Fast Red (Vector Labs, Burlingame, CA, USA) on each section for 1 min and subsequently rinsed the slides for 10 min with running tap water. Before mounting the slides with Entellan™ (Merck Darmstadt, Germany), slides were dehydrated using an increasing gradient of ethanol solutions and twice in xylene for 5 min.

### Histochemistry

Dio3 immunohistochemistry was performed as previously described (8). Briefly, paraformaldehyde-fixed and paraffin-embedded LV tissue sections (4 μm) were deparaffinized and rehydrated and exposed for 20 min to 0.02 M HCl. Epitope retrieval was performed by placing the sections at 93°C for 10 min in 10 M citrate buffer (pH 6.0). Sections were incubated with antibody 718 at 1:50 dilution at 37°C for 1 h and processed with Envision+ reagents (Dako, Glostrup, Denmark).

Polyclonal antibody 718 was raised in rabbit against the synthetic peptide KPEPEVELNSEGEEVP of the N-terminus of human DIO3 (amino acid residues 53–68). This antibody was kindly provided by Prof. D. Salvatore (University of Naples Federico II, Department of Molecular and Clinical Endocrinology and Oncology, Naples, Italy).

### Cell Culture and Western Blotting

Approximately 1.0 × 10^5^ COS-7 cells were seeded per well in 12-well plates in DMEM containing 10% FBS, 1% P/S. The next day, at 50–60% confluence, medium was refreshed. Cells were co-transfected with 100–400 ng full-length human DIO3 (wtD3) pcDNA3 vector (33). As a control, a mutant DIO3 expression vector was used that results in the replacement of the selenocysteine residue in the active center of the enzyme with cysteine (CysD3). Mimic-214-3p or negative miRNA control #1 was co-transfected at a final concentration of 500 nM (Ambion, Foster City, CA, USA). To isolate RNA and protein, medium was removed after 24 h, and cells were washed with PBS before being lysed with 50 μl lysis buffer (mirVana PARIS kit, Ambion, Foster City, CA, USA).

COS-7 cell lysates were separated on 15% SDS-PAGE gels and transferred to PVDF membrane by a semi-dry blotting system (Bio-Rad, Hercules, CA, USA). The membranes were cut into two at the 25 kDa weight marker and blocked with 5% (w/v) BSA in TBS containing 0.1% Tween (TBS-T) overnight at 4°C. Subsequently, the part of the membrane containing proteins larger than 25 kDa was incubated with antibody 677 (1:300), and the part of the membrane containing proteins smaller than 25 kDa was incubated with antibody 675 (1:1000) in 5% BSA in TBS-T for 2 h at room temperature. Membranes were washed three times for 10 min with TBS-T, followed by incubation with horseradish-peroxidase labeled goat anti-rabbit IgG in TBS-T (1:1000) for 1 h at room temperature and three times for 10 min with TBS-T. Bands were visualized using chemiluminescence (Amersham, Little Chalfont, UK) and scanned by LAS-3000 (Fujifilm Life Science, Stamford, CT, USA). Band intensity was quantified using Aida 4.21 (Raytest, Straubenhardt, Germany) and gel loading normalized to α-actinin (Sigma-Aldrich, St. Louis, MO, USA).

Polyclonal antibody 677 was raised in rabbit against the synthetic peptide RYDEQLHGARPRRV of the C-terminus of human DIO3 (amino acid residues 265–278) (33). Polyclonal antibody 675 was raised in rabbit against the synthetic peptide RRGKPEPEVELNS of the N-terminus of human DIO3 (amino acid residues 50–62). Both constructs and primary antibodies were kindly provided by Prof. T. Visser (Erasmus Medical Center, Rotterdam, The Netherlands). To correct for variations in transfection efficiency, DIO3 mRNA and protein levels were normalized to the mRNA level of the neomycin-resistance gene, expressed from the DIO3 pcDNA3 vectors.

### Dio3 Activity

Dio3 activity in LV homogenates was determined as described previously (33) with minor modifications. Reaction mixtures contained approximately 200,000 cpm of outer-ring-labeled T3 (3′-^125^I T3) and 0.25 mg protein in a final volume of 0.05 ml 0.1 M phosphate buffer (pH 7.2) containing 2 mM EDTA and 10 mM DTT. Mixtures were incubated for 60 min at 37°C and the reaction was stopped by addition of 0.05 ml ice-cold ethanol. After centrifugation, 0.075 ml of the supernatant was mixed with an equal volume of 0.02 M ammonium acetate (pH 4.0), and 0.1 ml of the mixture was applied to a 250 mm × 4.6 mm Symmetry C18 column connected to an Alliance HPLC system (Waters Chromatography Division, Millipore Corp., Milford, MA, USA) and eluted with a 15-min linear gradient of acetonitrile (28–42%) in 0.02 M ammonium acetate (pH 4) at a flow rate of 1.2 ml/min. Radioactivity in the eluate was monitored online using a Radiomatic A-500 flow scintillation detector (Packard, Meriden, CT, USA) and product formation by the inner-ring deiodination activity, i.e., (3′-^125^I T2) was quantified.

### Statistics

Data analysis was performed using Graphpad Prism version 6 (Graphpad, San Diego, CA, USA). Statistical significance was tested using a two-tailed Student *t*-test and accepted at *p* < 0.05.

## Results

### *In Silico* Analyses: Dio3 Is a Bona Fide Target of miR-214

**Figure 1:**
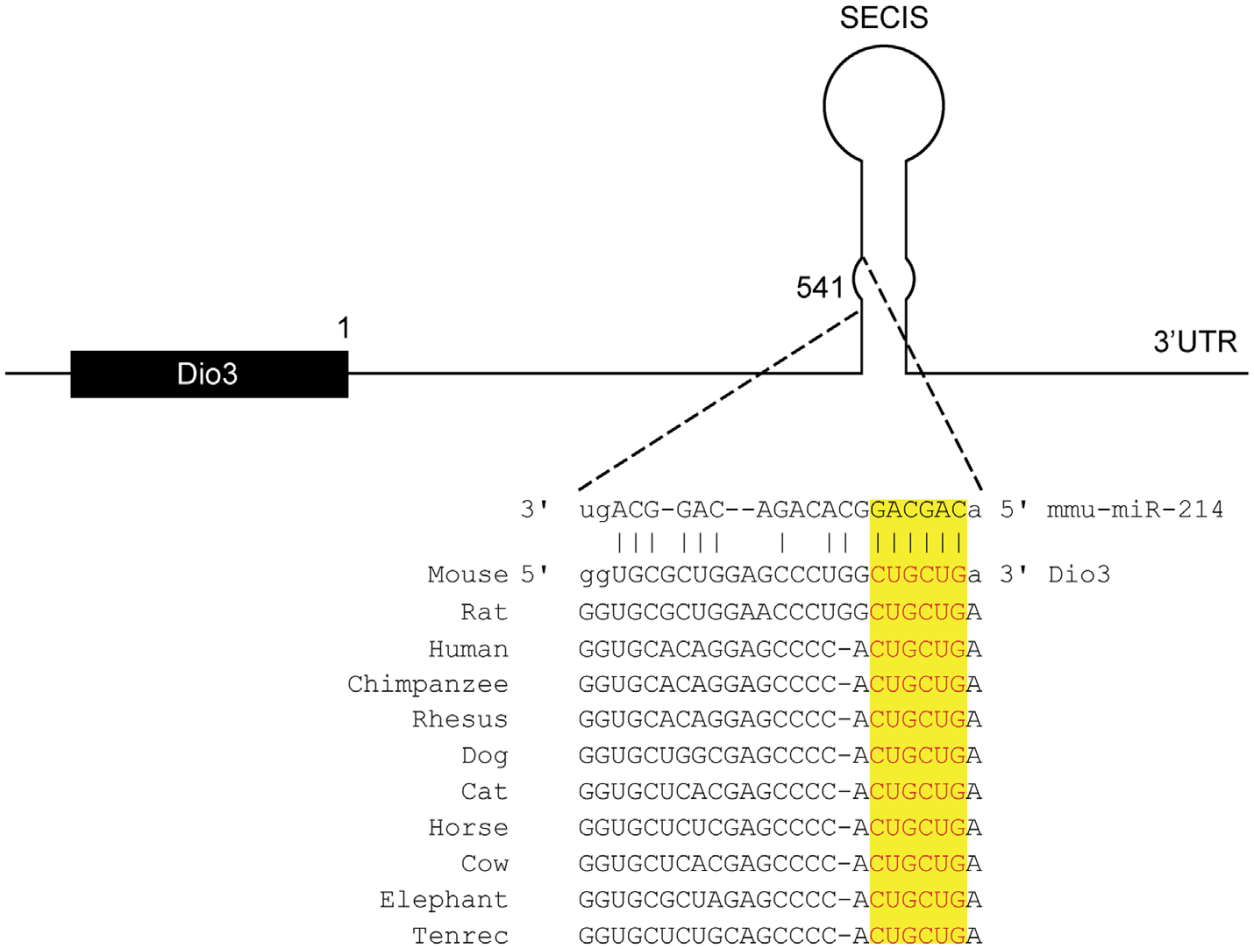
**Conserved miR-214 target site in the 3′-UTR of *Dio3***. *In silico* analysis predicts a target sequence for miR-214 in the stem of SECIS element of the 3′UTR of *Dio3* in mice, which is highly conserved among species. (Source: Targetscan and microRNA.org).

To identify potential miRNAs involved in regulating *Dio3* expression in the remodeling LV, we performed *in silico* analysis using four different prediction databases. MiR-214 was identified as having a high potential to target the 3′UTR of *Dio3*. A site complementary to the seed region of miR-214, which is highly conserved among species, was found to be located in the SECIS element, which is a secondary RNA structure (Figure 1).

### MiR-214 Expression Is Increased in the Remodeling LV of the Post-MI Heart

**Figure 2:**
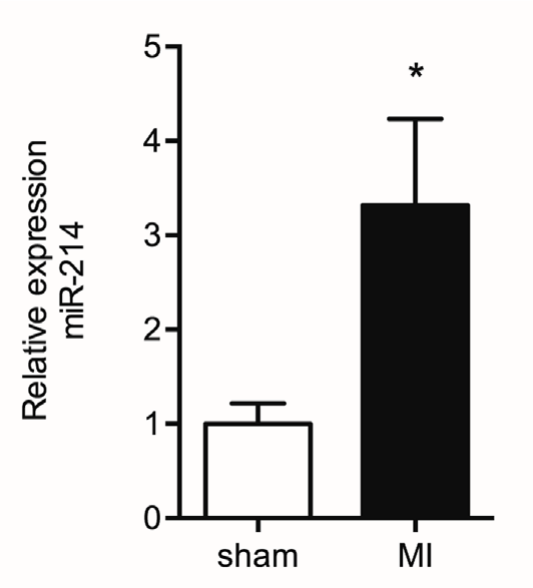
**Increased miR-214 expression in the post-MI LV**. Mir-214 expression levels were determined by quantitative RT PCR analysis in remote LV tissue from sham and MI animals isolated at day 7 post surgery. U6 snRNA expression levels were used as correction factor. Values are means ± SEM, *n* = 6, normalized to the sham level, *p < 0.05.

In a previous study, we identified miR-214 as a differentially regulated miRNA in the remodeling LV of the post-MI mouse heart compared to the LV of sham-operated mice, using TaqMan Megaplex array analysis of all 641 mouse miRNAs known at the time. Here, we validated the increased expression level of miR-214 in the post-MI heart using quantitative RT PCR analysis with U6 snRNA as correction factor, showing a significant threefold increase in miR-214 expression in the LV tissue 7 days post-MI surgery compared to sham-operated mice (Figure 2). Previous studies conducted by our group already established an approximately sixfold increased expression of *Dio3* mRNA using the same model (8, 25).

### Validation of Dio3 as a Target of miR-214

**Figure 3:**
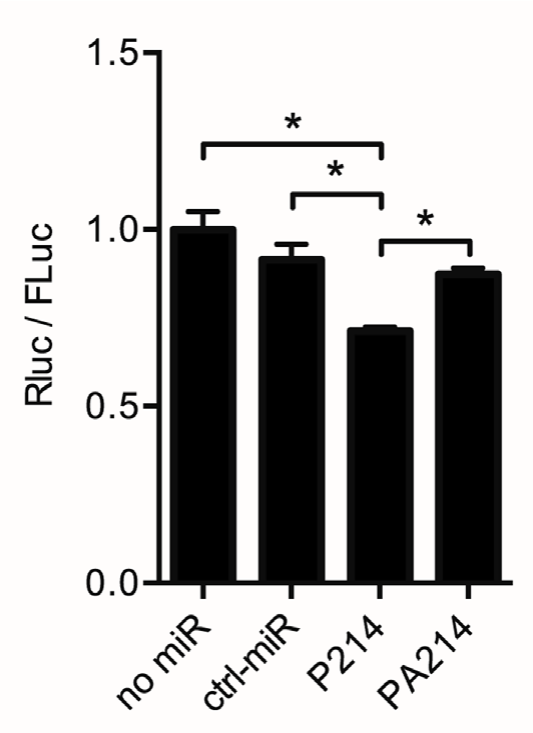
***Dio3* is targeted by miR-214**. A dual-luciferase construct containing the complete *Dio3* 3′UTR downstream of the *Renilla luciferase* gene was used to analyze the interaction between miR-214 and the 3′UTR of *Dio3*. Renilla luciferase activity (RLuc) is expressed relative to firefly luciferase activity (FLuc), correcting for differences in transfection efficiency. Four independent transfections were performed in duplicate. No-miR: control without co-transfection; ctrl-miR: + negative control/scrambled miR-1; P214: + Pre-miR-214; PA214: + Pre-miR-214 and Anti-miR-214. Values are means ± SEM, *n* = 4, normalized to the *RLuc/Fluc* ratio in the no-miR group, *p < 0.05.

To investigate whether miR-214 targets *Dio3*, we performed *in vitro* luciferase reporter experiments. A DNA-construct that contained the mouse *Dio3* 3′UTR inserted downstream of a luciferase reporter gene was transiently transfected into HEK293 cells. The results show a 30% reduction in *RLuc* activity when co-transfecting the reporter construct for 24 h with Pre-miR-214, relative to the “no-miR.” Compared to the condition with a control miRNA, this reduction was 22%. The addition of Anti-miR-214 to the transfection mix containing miR-214 abolished the repression (Figure 3). These luciferase reporter experiments support the *in silico* prediction that binding of miR-214 to the *Dio3* 3′UTR results in repression of Dio3 protein expression.

### Analysis of the Role of miR-214 in the Translation of DIO3 in Transfected COS Cells and the Involvement of the SECIS Element

Analysis of the 3′UTR of *Dio3* showed the presence of a highly conserved miR-214 target site in the SECIS element (Figure 1), which is present in both mouse and human DIO3. Since the SECIS element is essential for the incorporation of selenocysteine in DIO3, it led us to the question whether miR-214 might also interfere with *DIO3* translation at this level. The kind gift by prof. T. Visser of a DNA-construct containing the full-length human *DIO3* enabled us to investigate this possible interference in transfection experiments using cultured COS-7 cells.

Several studies showed that manipulation of the SECIS element results in increased levels of truncated DIO3. Therefore, COS-7 cells transfected with the plasmid containing the full-length human DIO3 (wtD3) together with mimic-214 or negative control miR-1 were analyzed for full-length (36 kDa) and truncated (18 kDa) DIO3 protein levels using western blotting. Expression levels were corrected using neomycin mRNA, transcribed from the same plasmid, as a control for transfection efficiency.

**Figure 4:**
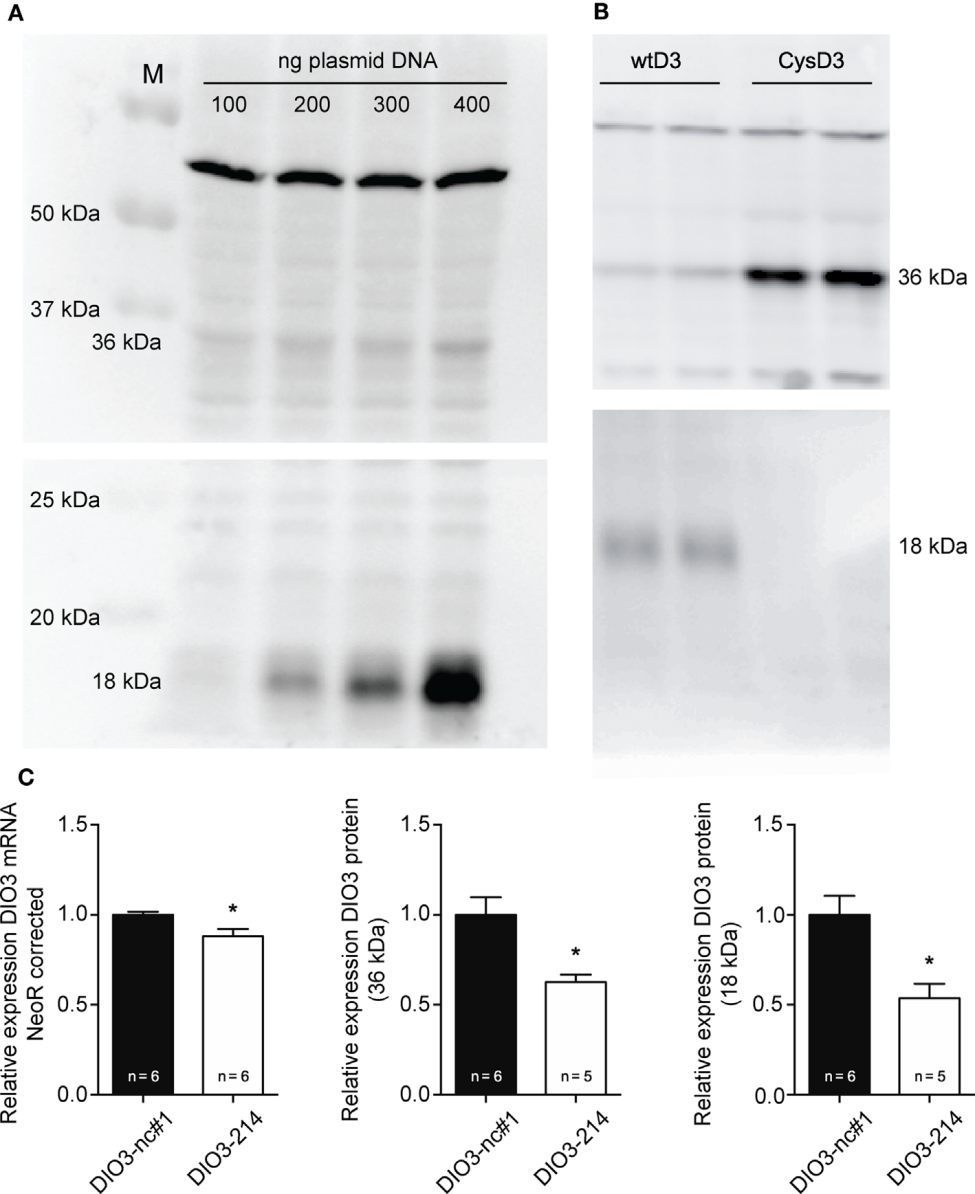
**Western blot analysis of DIO3 expression *in vitro***. **(A)** Transfection of COS-7 cells with increasing amounts of wtD3 plasmid confirmed the specificity of Ab 677 for full-length DIO3 (36 kDa) and of Ab 675 for truncated DIO3 protein (18 kDa). Equal amounts of protein were loaded. **(B)** COS-7 cells transfected with the CysD3 plasmid confirmed the expression of the truncated isoform of DIO3 in COS-7 cells transfected with the wtD3 plasmid, which was detected with Ab 675 (18 kDa) (lower blot; the upper blot is stained using Ab 677). **(C)** COS-7 cells were transfected with 100 ng of the wtD3 plasmid containing full-length human DIO3 together with either mimic-214 (DIO3 – 214) or Negative control miR-1 (DIO3-nc#1) at a final concentration of 500 nM. Addition of the negative control miR-1 did not significantly affect transfection efficiency (results not shown). Analysis of COS-7 cells transfected with empty pCDNA vector using Ab 677 and Ab 675 did not show bands at 36 kDa and 18 kDa, respectively, confirming antibody specificity (Figure S2 in Supplementary Material). Expression levels of the neomycin-resistance gene were analyzed using qPCR and used to normalize transfection efficiency. Values are means ± SEM, normalized to the value of the parameter in the DIO3-nc#1 group, *p < 0.05.

The specificity of Ab 677 and Ab 675 for the full-length and the truncated *DIO3*, respectively, was validated by transfecting COS-7 cells with increasing amounts of wtD3 plasmid DNA (Figure 4A). COS-7 cells transfected with expression vector encoding CysD3 (which exclusively yields full-length protein) confirmed the antibody specificity of Ab 675 against truncated DIO3 (Figure 4B). Co-transfecting the wtD3 plasmid with mimic miR-214 caused a ~13% reduction in mRNA expression when compared to cells co-transfected with negative control miR-1 (Figure 4C). Western blot analysis showed besides significantly reduced full-length DIO3 protein levels (~40%) (Figure 4C), reduced truncated DIO3 protein levels (~50%) (Figure 4C). However, the ratio of 36 over 18 kDa proteins remained unchanged (Figure S2 in Supplementary Material). This suggests that binding of miR-214 to the *DIO3* 3′UTR does not interfere with selenocysteine incorporation. The results indicate that binding of miR-214 results in a decreased *DIO3* mRNA stability, and although the reduction of DIO3 protein levels is larger than that of *DIO3* mRNA, it cannot be inferred from these data that *DIO3* translation efficiency is also affected by miR-214.

### Dio3 and miR-214 Are Co-Expressed in Adult Cardiomyocytes

**Figure 5:**
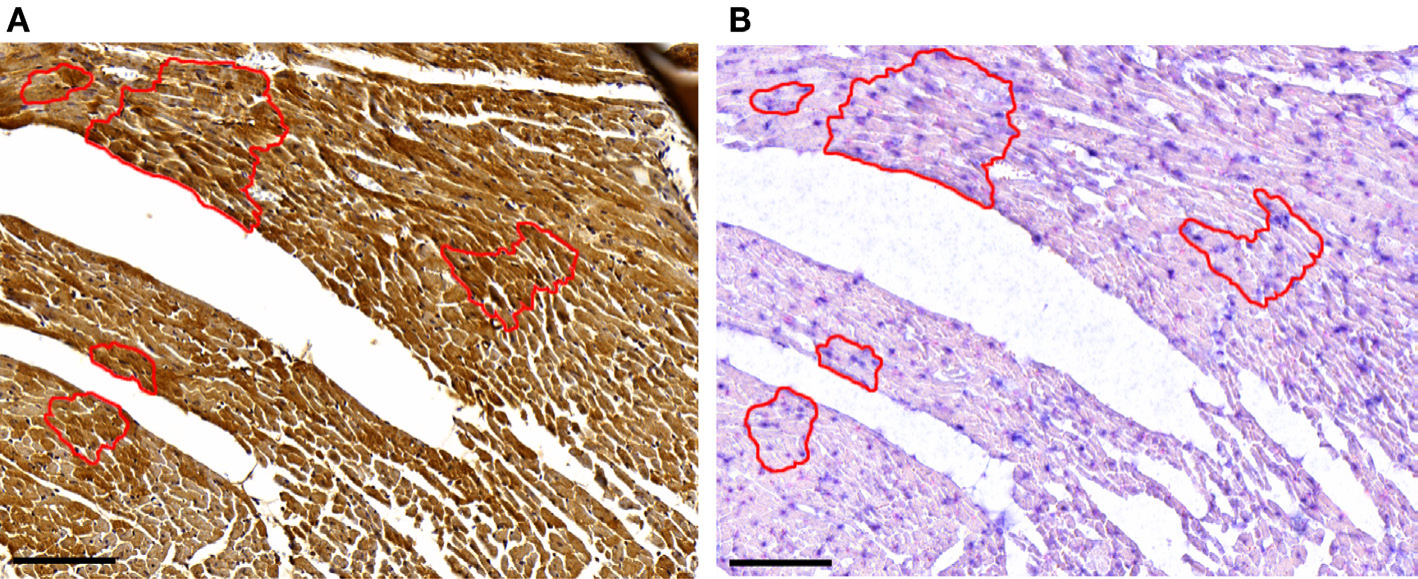
**Dio3 protein and miR-214 are co-expressed**. Representative images of immunohistochemical staining for Dio3 **(A)** and miR-214 *in situ* hybridization **(B)** in sequential sections of LV tissue 7 days post-MI. The indicated area shows a group of cardiomyocytes expressing both Dio3 protein and miR-214, with adjoining cells negative for both. Bar = 100 μM.

In a previous study, a heterogeneous, cardiomyocyte-specific expression pattern of Dio3 was observed in the post-MI LV (8). Based on the finding that miR-214 targets *Dio3*, we expected cardiomyocytes expressing a high level of Dio3 protein to have a low expression level of miR-214, and *vice versa*. Therefore, we performed miR-214 *in situ* hybridization and Dio3 immunohistochemistry on serial sections of post-MI LV tissue. A heterogeneous Dio3 expression pattern was again observed (Figure 5). Surprisingly, miR-214 *in situ* hybridization revealed a similar heterogeneous pattern, with the majority of miR-214 positive cardiomyocytes co-expressing Dio3 (Figure 5). This suggests either that Dio3 and 214-miRNA are coordinately, but independently upregulated, or that the expression of both is mechanistically linked.

### Dio3 Expression Precedes miR-214 Expression in the Post-MI LV

**Figure 6:**
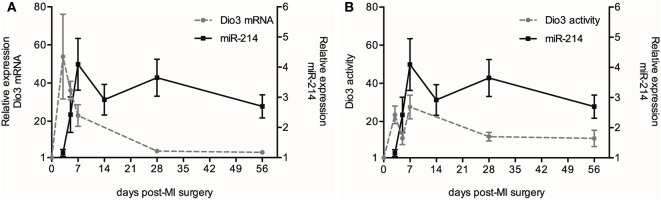
**Time course of miR-214 expression, *Dio3* mRNA and Dio3 activity after MI**. For the sake of clarity, the miR-214 data are shown in separate panels in combination with *Dio3* mRNA expression **(A)** and Dio3 activity **(B)**. The different parameters are expressed relative to their basal expression level determined in sham-operated mice at day 7. *Dio3* mRNA and Dio3 activity data from this set of samples were published in part before (8). Values are means ± SEM, miR-214 (*n* = 5), *Dio3* mRNA (*n*_3d_ = 7, *n*_5d_ = 6, *n*_7d_ = 16, *n*_28d_ = 10, *n*_56d_ = 16) and Dio3 activity (*n*_3d_ = 8, *n*_5d_ = 9, *n*_7d_ = 16, *n*_28d_ = 6, *n*_56d_ = 6).

Previous work conducted by our group revealed a time-dependent increase in both Dio3 expression and activity in the post-MI heart compared to sham animals (8). To further explore the possible interaction between *Dio3* and miR-214 expression, we analyzed miR-214 expression at 3, 5, 7, 14, 28, and 56 days post-MI surgery in the same set of LV samples that was previously used for analysis of *Dio3* mRNA expression and Dio3 activity (8). Figure 5 shows that miR-214 expression levels did not increase significantly until day 5 following MI compared to sham-operated mice, and reached a plateau at post-MI day 7, which was maintained for at least 56 days without significant changes. Time-course analysis showed that *Dio3* mRNA expression and Dio3 activity were already elevated at day 3 post-MI. Both *Dio3* mRNA and Dio3 activity subsequently decrease toward a steady elevated level 56 days post-MI compared to sham-operated mice (8) (Figures 6A,B, respectively). These data indicate that the increase in *Dio3* mRNA expression and Dio3 activity precede miR-214 induction, suggesting that any mechanistic link between the two involves an effect of Dio3 activity on the expression of miR-214.

### T3 Decreases Cardiac miR-214 Expression

**Figure 7:**
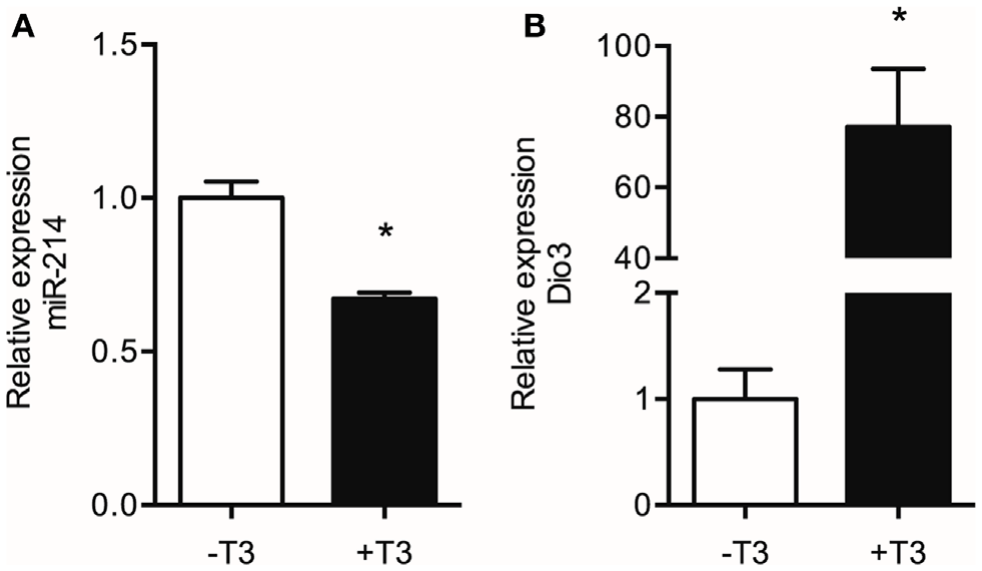
**Effect of T3 treatment on the levels of miR-214 and Dio3 mRNA**. Total RNA isolated from the LV of hypothyroid (−T3) and hypothyroid mice treated with T3 for three days (+T3) was analyzed by qPCR for miR-214 expression using U6 snRNA as correction factor **(A)**, and for Dio3 mRNA expression using Hprt as correction factor **(B)**. Values are means ± SEM, *n* = 6, normalized to the −T3 values, *p < 0.05.

The suggested regulation of miR-214 expression by increased *Dio3* expression would most likely be mediated by a local reduction in T3 levels induced by Dio3. In a previous study, we demonstrated that reduced T3 levels and reduced transcriptional activity of T3 accompany the Dio3 induction in the post-MI heart. Therefore, we examined the effect of T3 on miR-214 expression in LV tissue of hypothyroid (−T3) and hypothyroid mice treated with T3 for 3 days (+T3) mice. Exposure of the heart to high systemic levels of T3 resulted in a 50% reduction of miR-214 expression in the LV compared to the LV of hypothyroid mice (Figure 7). These findings suggest that reduced T3 levels may play a role in a negative feedback mechanism regulating *Dio3* expression that involves miR-214.

## Discussion

Several lines of evidence support the involvement of reduced TH-signaling in LV remodeling (14, 34, 35). The increased expression of the T3-degrading enzyme Dio3 in the failing heart provides the most likely explanation for the reduced TH-signaling and has emerged as a potential common denominator in the development of heart failure (7–11). However, the mechanism underlying the regulation of *Dio3* expression in the heart remains largely unclear. Since *Dio3* expression during development is known to be very precisely regulated in a spatiotemporal manner, miRNAs may be involved, as these regulators are known to play a role in fine-tuning of gene expression. The aim of the present study was to explore the possible involvement of miRNAs in the regulation of *Dio3* in the remodeling LV of the mouse after MI. We show that *Dio3* mRNA is a target of miR-214, and that this miRNA may play a role in a negative feedback mechanism regulating *Dio3* expression in the post-MI heart, thereby limiting the decrease of cardiac T3 levels.

*In silico* analysis revealed that of all miRNAs that were shown to be differentially regulated in the post-MI mouse heart (25), miR-214 was predicted to have the highest potential to target *Dio3*. This miRNA is suggested to play a role in the process of hypertrophy and heart failure in various models (21, 22, 27). Sequence analysis of the mouse *Dio3* 3′UTR predicted the presence of a miR-214 target site in the SECIS element, which was found to be highly conserved among species. Transfection experiments using a construct with a *Renilla luciferase* reporter gene containing the mouse *Dio3* 3′UTR demonstrated functional interaction of miR-214 with *Dio3* mRNA. The observed 30% reduction of reporter expression is comparable to the *in vitro* effects of miR-199b and miR-214, targeting *Dyrk1a* and *Ncx1*, respectively, which have been shown to modify cardiac performance (22, 24).

An interesting feature of the conserved miR-214 target site in the 3′UTR of *Dio3* is that it is located within the SECIS element, which is a secondary, double-stranded RNA structure. The SECIS element is responsible for the incorporation of selenocysteine (Sec) encoded by a UGA codon, which otherwise acts as a STOP codon (36). Impairment of the incorporation of Sec consequently results in a truncated form of DIO3 without deiodinase activity (33, 37, 38). We hypothesized that interference of miR-214 with the SECIS element of *DIO3* would result in increased levels of the truncated protein, and this was tested by analyzing the protein expression of full-length human DIO3 in COS-7 cells co-transfected with miR-214 using Ab 677 to detect the full-length 36 kDa DIO3 protein and Ab 675 to detect the 18 kDa truncated form of DIO3. Although miR-214 significantly affected DIO3 protein expression, the levels of both full-length and truncated protein were equally reduced, indicating that miR-214 does not interfere with the insertion of Sec. However, the substantially greater relative reduction of DIO3 protein expression compared to the effect on the *DIO3* mRNA level does suggest an additional effect of miR-214 on translation efficiency. Further research is needed to elucidate the mechanism of interaction of miR-214 in regulating DIO3 expression.

Having confirmed the suppressive effect of miR-214 on *Dio3* expression, it was unexpected that the expression of miR-214 was increased in the post-MI mouse heart, given the marked upregulation of cardiac Dio3 activity in this model (8, 25). To explore the possible interplay between miR-214 and *Dio3*, their expression in cardiomyocytes was studied in sections of LV tissue. Immunohistochemical analysis of the post-MI LV confirmed the previously found heterogeneous expression of Dio3 protein (8). Also miR-214 exhibited a heterogeneous expression pattern, and analysis of serial sections indicated that Dio3 and miR-214 were co-expressed in cardiomyocytes, suggesting either that *Dio3* and miR-214 are independently upregulated or that the expression of miR-214 and *Dio3* expression is mechanistically linked. The latter option was supported by comparison of the temporal changes in miR-214 expression in the remodeling LV from the onset of MI up to 56 days later, with those of *Dio3* mRNA and Dio3 activity as previously determined in the same samples. This showed that the increase of *Dio3* mRNA levels and Dio3 activity [data from Ref. (8)] preceded the increase in miR-214 expression. While *Dio3* mRNA levels and Dio3 activity were already increased to high levels at days 3–5 post-MI, miR-214 expression was still only modestly elevated. Furthermore, the expression of miR-214 expression reached a maximum level at day 7 post-MI, by which time the *Dio3* mRNA and Dio3 activity levels appeared to go down, but still remained elevated compared to sham. The expression of miR-214 remained, however, unchanged up to at least 56 days post-MI. The increase of miR-214 over time is in line with previous results in a mouse MI model (26), showing increasing miR-214 expression levels over time in remote myocardium (26).

**Figure 8:**
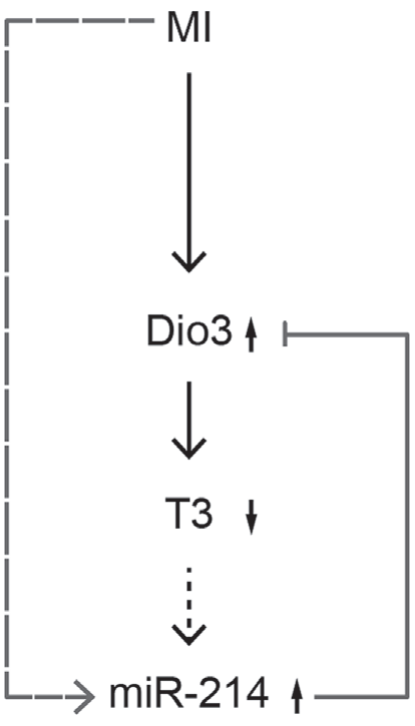
**Proposed mechanism of Dio3 regulation by miR-214**. MI-induced cardiac stress results in upregulation of Dio3, leading to a reduced cardiac level of T3. The decreased level of T3 stimulates the expression of miR-214, which adds to the effects of other MI-induced regulatory pathways on miR-214 expression (the dotted arrow indicates that the relationship between T3 and miR-214 is inferred from indirect evidence). Increased miR-214 expression in turn decreases Dio3 expression, thereby dampening the reduction of T3 levels by Dio3. In this way, a negative feedback loop is created, possibly aimed at protecting the adult cardiomyocyte from the adverse effects of excessively low levels of T3.

These data suggest a mechanistic link between miR-214 and Dio3 expression, which could involve the Dio3-mediated reduction of cardiac T3 levels. This implies that expression of miR-214 is negatively regulated by T3, and this was confirmed by the finding that treatment of hypothyroid mice with T3 markedly suppressed miR-214 expression levels in the LV. This may be a direct effect on transcription or processing of miR-214, but perhaps more likely involves components of the signal transduction pathways that drive physiological rather than pathological hypertrophy, and which are triggered by the effect of T3 on cardiac hemodynamic load (14, 30). *Dio3* mRNA expression showed a reciprocal response, being higher in the LV of T3-treated mice compared to the LV of hypothyroid mice. This is in line with previous findings of stimulation of *Dio3* expression by high levels of T3 (39, 40). These studies provided evidence for a T3-dependent threefold induction of *Dio3* expression after 24 h. Although suggestive of a direct transcriptional effect of T3, the mechanism of stimulation remained unclear. Our data suggest that in the post-MI heart, *Dio3* expression may at least be additionally modulated by T3 via changes in miR-214 expression. Taken together, the result of the present study support the involvement of miR-214 in a negative feedback mechanism regulating Dio3 expression. The proposed sequence of events is depicted in Figure 8. In the post-MI heart, *Dio3* expression is induced, possibly as part of the fetal gene program. This results in an increase in the local conversion of T3 to the inactive metabolite T2. We hypothesize that the documented local decrease of T3 levels stimulates the expression of miR-214, which dampens *Dio3* expression. The relevance of this mechanism lies in the prevention of an excessive decrease of cardiac T3 levels and the associated changes in expression of genes that are critical for cardiac function (14). MiR-214 expression is likely to be regulated by other mechanisms associated with the remodeling process in addition to the decreased T3 levels. It was shown that Hif-1α, which is a key determinant of progression in the remodeling LV (41), activates the locus dinamin 3 opposite site (Dnm3os) harboring miR-214 (27). Upregulation of Hif-1α after MI is generally observed in the infarct or peri-infarct zone, but Hif-1α-dependent transcription activity was found in the remote LV at day 5 post-MI (Pol, unpublished results), which may contribute to miR-214 upregulation.

In conclusion, this study first shows that miR-214 is able to target both the human and the mouse 3′UTR of *DIO3*, affecting mRNA and protein expression. Second, the results indicate a novel negative feedback mechanism regulating Dio3 expression in the post-MI mouse heart, in which a Dio3-mediated decrease of T3 levels results in increased expression miR-214, which subsequently reduces Dio3 expression. This mechanism may, therefore, be aimed at restoring, or limiting the reduction of T3 levels in the post-MI heart (Figure 8).

## Author Contributions

RJ, CO, WP and WS designed the study. RJ, AM, AvM, ED and WS designed the experiments. RJ, MZ and AvM performed the experiments. RJ analyzed the data. RJ, AM and WS wrote the manuscript. WS and AM supervised the study.

## Conflict of Interest Statement

The authors declare that the research was conducted in the absence of any commercial or financial relationships that could be construed as a potential conflict of interest.
