# Supplementary material for: MicroRNA 214 Is a Potential Regulator of Thyroid Hormone Levels in the Mouse Heart Following Myocardial Infarction, by Targeting the Thyroid-Hormone-Inactivating Enzyme Deiodinase Type III
Source: Front Endocrinol (Lausanne). 2016 Mar 9;7:22. doi: 10.3389/fendo.2016.00022 (PMC4783388; doi:10.3389/fendo.2016.00022)
Supplement: Supplementary file 1 [file Table_1.doc]

**9. Supplementary Material**

Table SM1.Primersets used to analyze mRNA expression by qPCR.

|  | Forward primer 5’-3’ | Reverse primer 5’-3’ |
| --- | --- | --- |
| *Hprt* | TCCCTGGTTAAGCAGTACAGCC | CGAGAGGTCCTTTTCACCAGC |
| *Myh6* | GACCAGGCCAATGAGTACCG | GCCTAGCCAACTCCCCGTTC |
| *Myh7* | CGCTCCACGCACCCTCACTT | GTCCATCACCCCTGGAGAC |
| *Anf* | CGAAGATCCAGCTGCTTCGG | TTCGGTACCGGAAGCTGTTG |
| *Dio3* | CGCTCTCTGCTGCTTCAC | TCTCCTCGCCTTCACTGTTGA |
