# Supplementary figures and images for: MicroRNA 214 Is a Potential Regulator of Thyroid Hormone Levels in the Mouse Heart Following Myocardial Infarction, by Targeting the Thyroid-Hormone-Inactivating Enzyme Deiodinase Type III
Source: Front Endocrinol (Lausanne). 2016 Mar 9;7:22. doi: 10.3389/fendo.2016.00022 (PMC4783388; doi:10.3389/fendo.2016.00022)

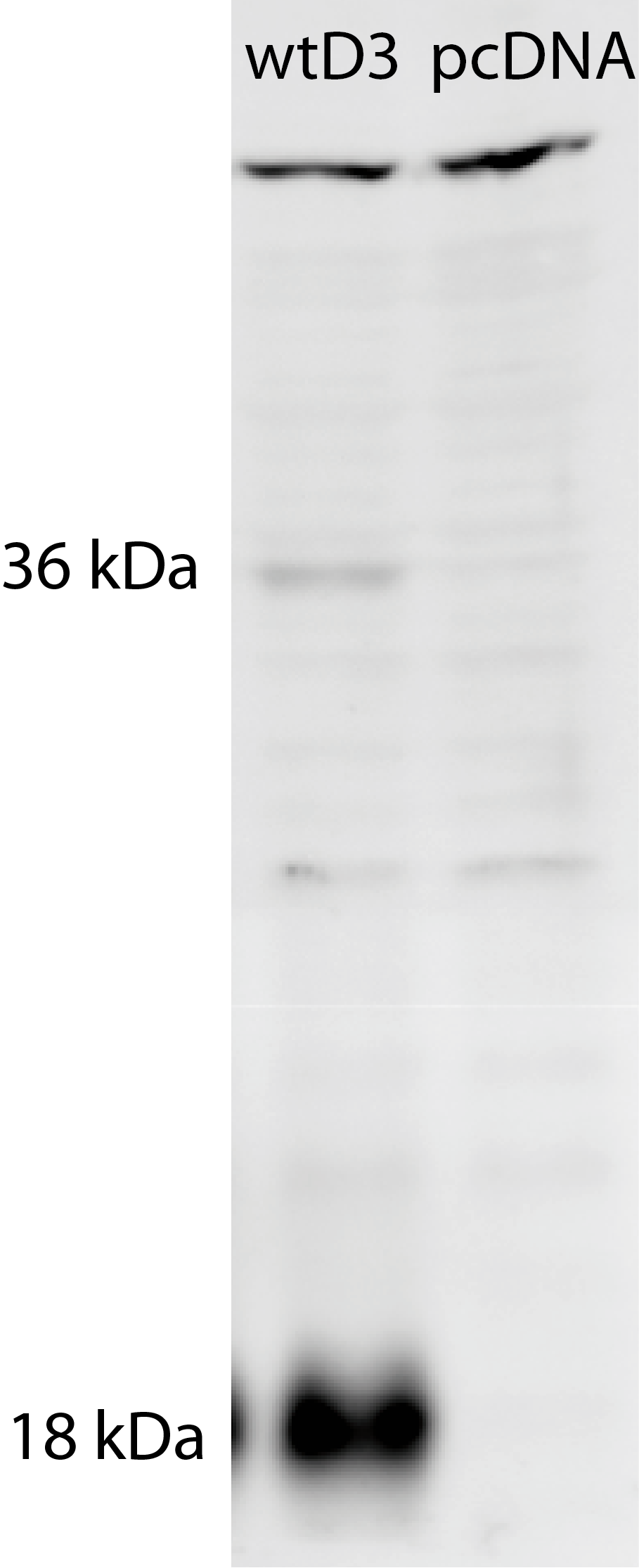

Supplement: Supplementary file 2 [file Image_1.TIF]

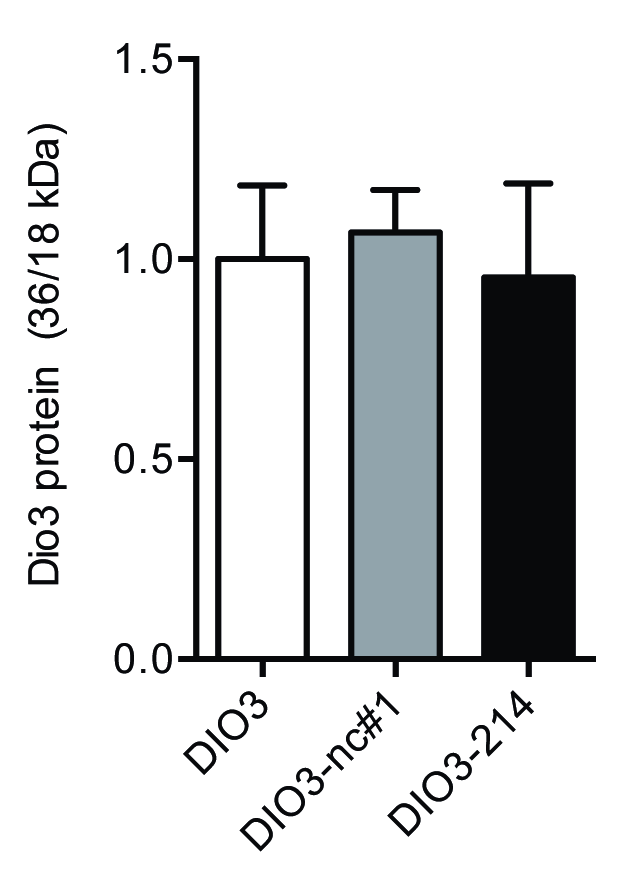

Supplement: Supplementary file 3 [file Image_2.TIF]
